# Kaempferol-7-O-Glucoside Ameliorates Atopic Dermatitis via the TSLP-Mediated JAK2/STAT5 Signaling Axis

**DOI:** 10.3390/ph19040580

**Published:** 2026-04-04

**Authors:** Xingmei Lan, Jing Liu, Yijie Shi, Yonghua Zhou, Cheng Yang, Bingtian Zhao

**Affiliations:** 1 Key Laboratory of Synthetic and Biological Colloids, Ministry of Education, School of Chemical and Material Engineering, Jiangnan University, Wuxi 214122, China; lanxingmei999@163.com (X.L.); 1053230304@stu.jiangnan.edu.cn (Y.S.); 2 Key Laboratory of National Health Commission on Parasitic Disease Control and Prevention, Jiangsu Provincial Key Laboratory on Parasite and Vector Control, Jiangsu Institute of Parasitic Diseases and Public Health Research Center of Jiangnan University, Wuxi 214064, China; toxo2001@163.com

**Keywords:** atopic dermatitis, TSLP, flavonoids, JAK/STAT signaling pathway, structure–activity relationship

## Abstract

**Background/Objectives:** Thymic stromal lymphopoietin (TSLP) is central to the pathogenesis of atopic dermatitis (AD) and a promising therapeutic target. However, developing small-molecule TSLP inhibitors is challenging due to the difficulty in disrupting the TSLP-TSLPR interface. This study aimed to explore naturally sourced blockers of the TSLP-TSLPR interaction and identify novel candidate compounds for AD treatment. **Methods:** HuT78 cells were stimulated with PMA, ionomycin, and TSLP to establish an AD model. Inflammatory cytokines were measured by qRT-PCR and ELISA. JAK/STAT signaling was analyzed by Western blot. In female BALB/c mice, DNCB-induced AD-like skin lesions were topically treated with test compounds, followed by histopathological and immunohistochemical assessment. **Results:** Eight compounds were screened, and their key structural features were elucidated via structure–activity relationship (SAR) analysis. Among them, kaempferol-7-O-glucoside (K-7-G) emerged as the most potent candidate. It interfered with the TSLP-TSLPR interaction, selectively inhibited TSLP-mediated JAK2/STAT5 phosphorylation, and significantly downregulated IL-4 (*p* < 0.0001) and IL-13 (*p* < 0.001) levels. Topical application of 1% K-7-G significantly alleviated AD-like symptoms in a mouse model, decreasing dorsal skin thickness, dermatitis score, and scratching frequency while restoring the expression of filaggrin, loricrin, and occludin (*p* < 0.0001). Meanwhile, it significantly reduced key inflammatory mediators in a concentration-dependent manner, including TSLP, IL-4, IL-13, TNF-α, IFN-γ, and IgE. **Conclusions:** This study demonstrates that K-7-G is a novel natural TSLP inhibitor capable of blocking the TSLP-TSLPR signaling pathway and effectively improving AD symptoms. Further research may explore its therapeutic potential in other inflammatory diseases.

## 1. Introduction

Atopic dermatitis (AD), a long-lasting inflammatory skin disorder, is defined by intense itching and dry skin [1]. Previous studies have shown that the worldwide prevalence of AD is estimated at 15–20% among children and up to 10% in adults [2]. AD pathogenesis stems from the combined effects of inherited risk factors, weakened skin barrier function, and dysregulated Th2-mediated inflammatory pathways, collectively driving disease progression [3]. For decades, topical corticosteroids and calcineurin inhibitors have remained the mainstay of AD therapy. However, prolonged administration of the relevant intervention correlates with notable adverse reactions. Corticosteroids can cause localized skin atrophy, whereas the tumor risk of long-term topical calcineurin inhibitors remains unclear [4,5]. This situation underscores an unmet need for safer, more tolerable therapies.

Thymic stromal lymphopoietin (TSLP) acts as a critical mediator of AD pathophysiology and is produced primarily by epithelial cells [6]. Functioning as an epithelial-derived alarm molecule, TSLP selectively binds to the heterodimeric receptor composed of TSLPR and IL-7Rα subunits. This interaction triggers the phosphorylation of STATs mediated by JAK1 and JAK2, thereby regulating the transcription of downstream target genes, including IL-4, IL-5, IL-9, and IL-13 [7]. The JAK/STAT pathway enhances inflammatory responses not only by directly prompting innate immune cells to secrete pro-inflammatory cytokines but also by its critical function in DC activation, thereby facilitating the differentiation of naive CD4^+^ T cells into Th2 cells [8]. As a key mediator, the JAK/STAT pathway influences the onset of various inflammatory diseases by regulating immune functions and contributing to pathological processes. Meanwhile, the produced cytokines collectively downregulate skin barrier proteins (e.g., filaggrin and loricrin) and tight junction proteins (e.g., occludin) in keratinocytes, exacerbating epidermal barrier dysfunction. Given that TSLP orchestrates multiple pathogenic pathways in AD, from initiating early inflammation and regulating Th2 cell differentiation to aggravating barrier impairment, blocking the TSLP-TSLPR interaction has emerged as a promising strategy to target AD [9,10].

Indeed, therapies that target TSLP signaling, such as biological agents, have shown promising efficacy in alleviating the symptoms of AD. However, these agents are often costly and carry potential long-term side effects, limiting their accessibility and utility [11]. This has led to a rising demand for alternative treatment strategies that are affordable, well-tolerated, and capable of targeting multiple pathogenic axes of AD. In light of these limitations, flavonoids have gained increasing attention due to their diverse bioactivities and low toxicity [12]. Currently, accumulating evidence supports the regulatory effects of flavonoids on the TSLP signaling pathway. For instance, studies have demonstrated that quercetin significantly inhibits TSLP production in mouse models of allergic airway inflammation [13]. Kaempferol exerts a significant therapeutic effect on dermatitis models, with mechanisms including the inhibition of TSLP synthesis, the promotion of epidermal barrier protein expression, a reduction in IL-4/IL-13 secretion, and the alleviation of oxidative stress [14]. Furthermore, baicalein is identified as the pioneering small-molecule inhibitor targeting TSLP, and it successfully inhibits eosinophil infiltration in mouse models induced by house dust mites/ovalbumen, laying the groundwork for the development of its analogs as new anti-allergy therapeutics [10]. However, existing studies face two major technical bottlenecks. First, the reliance on intraperitoneal injection fails to precisely target cutaneous lesions and compromises efficacy due to low local drug concentration [15]. Second, related research is still in its preliminary stages, lacking a systematic strategy to identify effective flavonoid inhibitors of the TSLP/JAK/STAT pathway or a comprehensive structure–activity relationship (SAR) analysis.

Given the pivotal role of TSLP in driving AD-related inflammatory responses and the current lack of suitable natural product-derived TSLP inhibitors, this study aimed to identify a highly potent natural TSLP inhibitor for AD treatment. Through computational screening, experimental validation, and SAR analysis, we elucidated that K-7-G harbors the key structural features responsible for TSLP pathway inhibition. Finally, the therapeutic efficacy of K-7-G was evaluated in an AD mouse model, and its underlying mechanism as a novel TSLP inhibitor was delineated. This study demonstrates that K-7-G is a highly promising small-molecule TSLP inhibitor, laying a critical theoretical foundation for the development of flavonoid-based therapeutic strategies targeting TSLP-mediated diseases.

## 2. Results

### 2.1. Targeted Screening of Flavonoids Against the TSLP-TSLPR Interface

**Figure 1:**
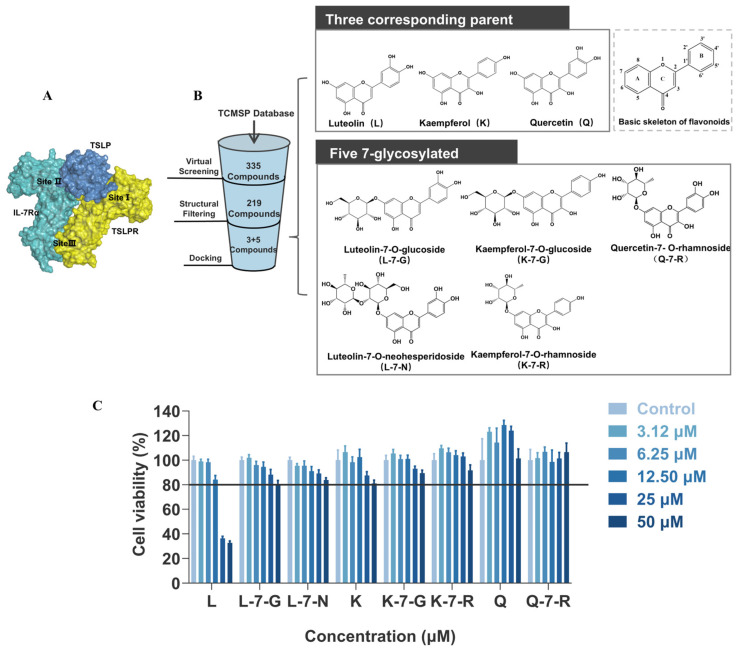
Molecular docking-based screening and cell viability assay. (**A**) Schematic diagram of the ternary complex structure. (**B**) Workflow for screening TSLP-targeted flavonoid candidates. (**C**) Cell viability of HuT78 cells treated with different compounds by CCK-8 assay (*n* = 4).

To explore potential strategies for disrupting the TSLP-TSLPR interface, the structural basis of TSLP receptor complex assembly was examined. Previous research has shown that TSLP ternary signaling complex assembly displays orderliness and cooperativity, where Site I (TSLP:TSLPR) acts as a core pivotal step [16]. TSLP exhibits a strong affinity for binding to the TSLPR on the cell surface, creating a binary complex that is stabilized by particular interactions and electrostatic complementarity (Site I). This site is essential for the subsequent high-affinity recruitment of IL-7Rα to form Site II (TSLP:IL-7Rα), which together assemble the T-shaped ternary complex. TSLP also facilitates the bridging of the two receptors, forming Site III (TSLPR:IL-7Rα) and triggering subsequent downstream signaling (Figure 1A) [17]. The affinity between TSLP and IL-7Rα was enhanced by about three orders of magnitude at site I, emphasizing its critical function in the mechanism [7]. Building on this structural insight, the structure of the ternary complex was employed to define Site I as the target interface for screening small-molecule inhibitors, with the aim of disrupting TSLP:TSLPR complex formation to block TSLP-mediated signaling (Figure 1B).

Eight compounds were finally successfully identified by employing molecular docking, comprising three parent compounds (L, K, and Q) and five flavone glycosides (L-7-G, L-7-N, K-7-G, K-7-R, and Q-7-R). The binding energy of the parent compounds was less than −6.0 kcal/mol, while the five flavonoid glycosides had binding energies lower than −7.0 kcal/mol (in Table S1). This indicates that glycosylation significantly enhances the binding affinity to the target. These compounds were subjected to subsequent testing in the TSLPR-expressing HuT78 cell line. Cell viability assay results showed that five flavonoid glycosides and their parent compounds K and Q exhibited weak cytotoxicity. In contrast, the cytotoxicity of compound L was significantly stronger at 25 and 50 μM. Accordingly, the maximum concentration used in subsequent experiments was set to 12.50 μM (Figure 1C).

### 2.2. DEGs Screening and Functional Enrichment Analysis

#### 2.2.1. Screening of DEGs in the Model of Th2-Type Inflammation

**Figure 2:**
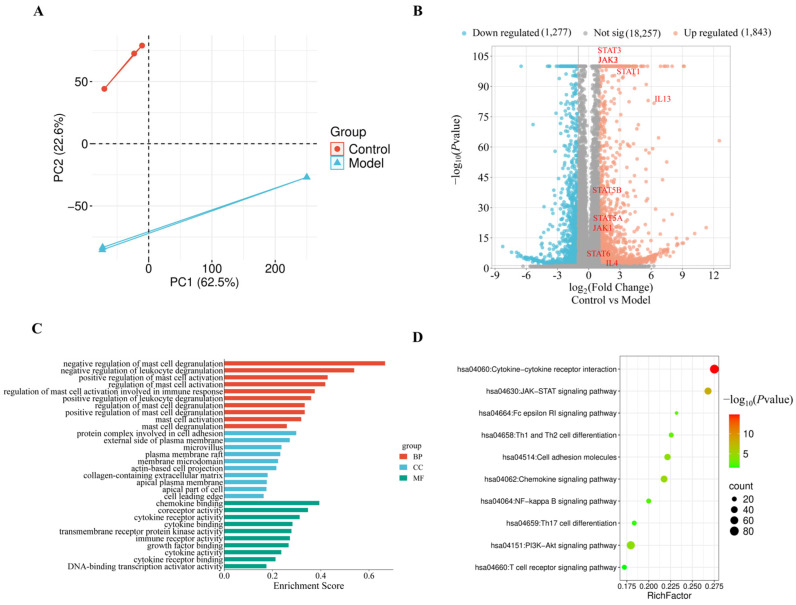
Transcriptomic analysis of DEGs between the control and model groups (*n* = 3). (**A**) PCA plot. (**B**) Volcano plot illustrating upregulated and downregulated DEGs. (**C**) Bar chart of GO enrichment results across BP, CC, and MF categories. (**D**) Bubble plot of KEGG analysis. Data are shown as mean ± SEM. For the volcano plot (**B**), DEGs were determined by the Wald test (DESeq2) with Benjamini–Hochberg FDR correction.

For clarifying the molecular mechanisms involved in disease progression and determining crucial inflammatory pathways, transcriptome sequencing was performed on normal HuT78 cells and TSLP-induced HuT78 cells, followed by the analysis of principal component analysis (PCA) and differentially expressed genes (DEGs). PCA showed clear separation between the two groups, with high intra-group consistency, indicating significant transcriptional heterogeneity (Figure 2A). Using thresholds of |log_2_FC| > 1 and FDR < 0.05, a total of 3120 DEGs were identified, including 1843 upregulated and 1277 downregulated in the TSLP-induced group (Figure 2B). Consistently, multiple pathway components were transcriptionally upregulated, including all three JAK kinases (JAK1, JAK2, and JAK3), five STAT family members (STAT1, STAT3, STAT5A, STAT5B, and STAT6), and type 2 cytokines (IL-4 and IL-13). The results indicate that TSLP mediates its regulatory effects through activation of the JAK/STAT pathway and upregulation of downstream type 2 cytokines.

#### 2.2.2. GO and KEGG Enrichment Analysis of DEGs

For a deeper exploration of the biological significance of DEGs, GO and KEGG were carried out. This analysis identified 30 significantly enriched GO terms, comprising 10 BP, 10 CC, and 10 MF categories (Figure 2C). Within BP, DEGs were primarily engaged in immune-associated processes, including negative regulation of mast cell degranulation and leukocyte degranulation, positive regulation of mast cell activation, and regulation of mast cell activation. In the CC category, DEGs were predominantly localized to the outer plasma membrane. In the MF category, they were largely associated with chemokine binding, coreceptor activity, cytokine receptor activity, and cytokine binding.

KEGG pathway analysis results suggested that DEGs in the model group were significantly enriched in several key pathways, including cytokine–cytokine receptor interaction, JAK/STAT signaling, FcεRI signaling, and Th1/Th2 cell differentiation. Notably, these pathways converge on Th2-driven immune regulation, reinforcing the central role of IL-4 and IL-13 upregulation via the JAK/STAT pathway in AD pathogenesis (Figure 2D).

### 2.3. Effects of Eight Flavonoids on IL-4 and IL-13 Expression in HuT78 Cells

**Figure 3:**
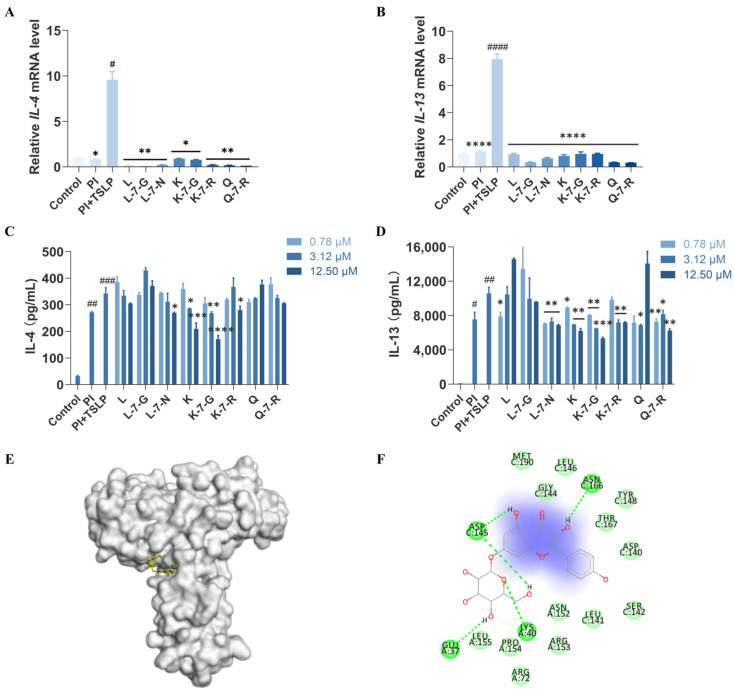
Screening of eight flavonoid compounds in HuT78 cells. (**A**,**B**) qRT-PCR analysis of IL-4 and IL-13 mRNA (*n* = 3). (**C**,**D**) IL-4 and IL-13 were quantified by ELISA (*n* = 3). (**E**) 3D docking model of K-7-G with TSLP and TSLPR. (**F**) 2D docking representation of K-7-G. Data are presented as mean ± SEM. (**A**,**B**) Statistical significance was determined by one-way ANOVA with Dunnett’s test; (**C**,**D**) Two-way ANOVA with Dunnett’s test. ^#^ *p* < 0.05, ^##^ *p* < 0.01, ^###^ *p* < 0.001, and ^####^ *p* < 0.0001 vs. Control group; * *p* < 0.05, ** *p* < 0.01, *** *p* < 0.001, and **** *p* < 0.0001 vs. PI + TSLP group.

To investigate the therapeutic potential of flavonoids as inhibitors of the TSLP signaling pathway, we assessed TSLP-induced changes in IL-4 and IL-13 expression in HuT78 cells. The addition of PI + TSLP led to a substantial rise in IL-4 and IL-13 mRNA expression (*p* < 0.05 or *p* < 0.0001), whereas pre-treatment with the eight flavonoids considerably suppressed this effect (Figure 3A,B). Meanwhile, consistent with the mRNA results, a significant elevation in IL-4 and IL-13 protein levels was observed in TSLP-stimulated cells after 36 h (*p* < 0.001 or *p* < 0.01), compared to the unstimulated controls (Figure 3C,D). Against this background of enhanced cytokine secretion, all tested flavonoids attenuated cytokine release to varying degrees. Among them, K-7-G exerted the strongest and most distinct concentration-dependent inhibition: at 12.5 μM, it reduced IL-4 levels by 50.2% (*p* < 0.0001) and IL-13 levels by 49.5% (*p* < 0.001) compared with the model group. Next, K exhibited moderate inhibitory activity, decreasing IL-4 by 39.0% (*p* < 0.001) and IL-13 by 41.4% (*p* < 0.01). K-7-R, Q-7-R, and L-7-N exhibited weaker but measurable inhibitory effects, whereas Q, L, and L-7-G showed minimal activity and were largely ineffective. Collectively, these comparisons established the following potency ranking: K-7-G > K > K-7-R ≈ Q-7-R ≈ L-7-N > Q ≈ L ≈ L-7-G, highlighting K-7-G as the most promising candidate.

The glucosyl group linked to the C-7 position of K-7-G forms multiple specific hydrogen bonds with polar residues on the protein surface (e.g., GLU37, LYS40, and ASP145) via its polyhydroxyl structure (-OH) (Figure 3E,F). These additional hydrogen bond sites provided by the glucosyl group significantly enhance the polar interactions between the compound and the protein. This facilitates the tight binding of K-7-G to TSLP-TSLPR, thereby effectively blocking their interaction. The kaempferol aglycone moiety forms hydrophobic interactions with LEU141, LEU146, and TYR148, thus indicating that the predicted binding conformation is further stabilized. The docking diagrams of other compounds are shown in Figure S1.

### 2.4. Compounds Inhibit TSLP-TSLPR Interaction

**Figure 4:**
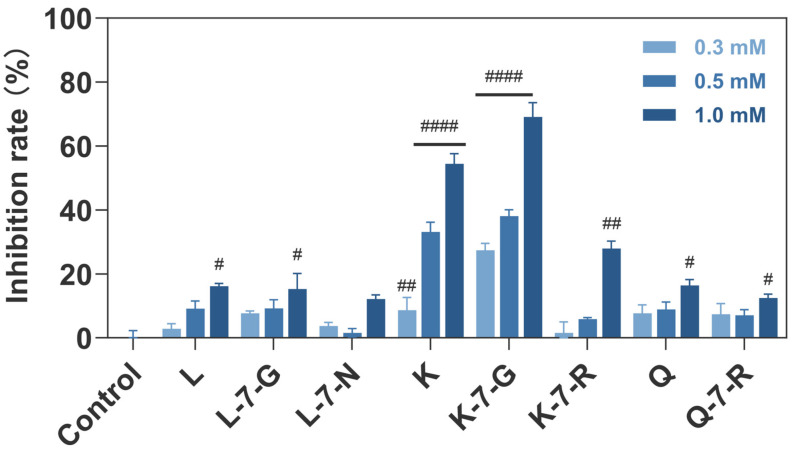
Inhibitory effects of eight flavonoid compounds on TSLP-TSLPR interaction (*n* = 3). Data are presented as mean ± SEM. Two-way ANOVA with Dunnett’s test was used to compare each treatment group with the Control group. ^#^ *p* < 0.05, ^##^ *p* < 0.01, and ^####^ *p* < 0.0001 vs. Control group.

To directly evaluate the ability of flavonoids to inhibit TSLP-TSLPR binding, a competitive ELISA assay was performed [18]. K-7-G exhibited the most potent inhibitory activity, with 69.1% inhibition at 1.0 mM (Figure 4). In contrast, K showed moderate inhibitory activity (54.4% inhibition), while all other tested compounds displayed less than 30.0% inhibition. This is consistent with the aforementioned results that K-7-G exerted the best effect in HuT78 cells, followed by K. By blocking this critical receptor–ligand binding, K-7-G may attenuate TSLP-mediated signaling and thereby suppress downstream inflammatory responses.

### 2.5. Effects of Three Compounds on the JAK/STAT Pathway

**Figure 5:**
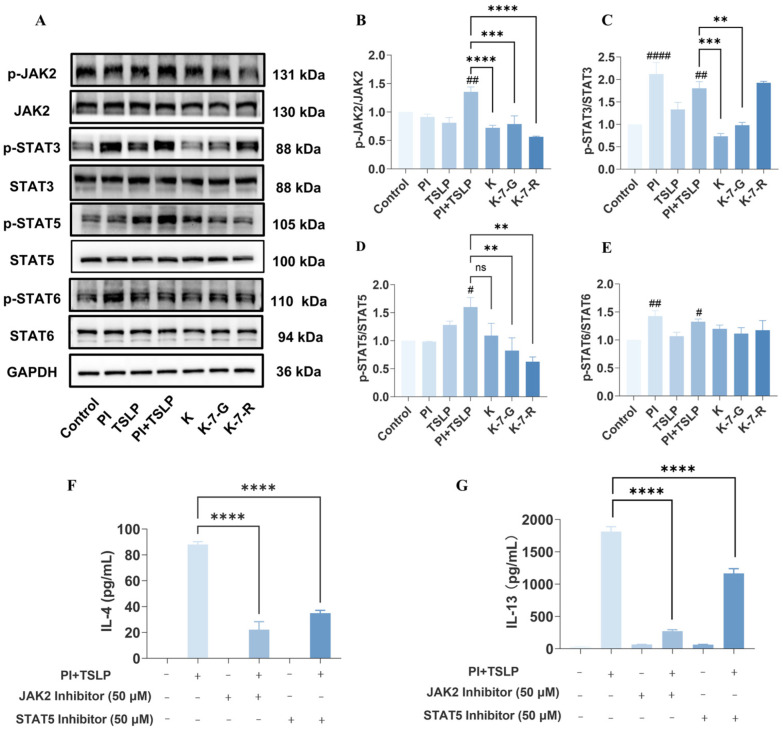
Inhibition of TSLP signaling by K, K-7-G, and K-7-R. (**A**) Phosphorylation levels of JAK2, STAT3, STAT5, and STAT6 were detected (*n* = 3). (**B**–**E**) Relative protein intensities were quantified by densitometry using ImageJ 1.52i software (*n* = 3). (**F**,**G**) IL-4 and IL-13 were measured (*n* = 3). Data are presented as mean ± SEM. One-way ANOVA with Tukey’s test was used for multiple comparisons. ^#^ *p* < 0.05, ^##^ *p* < 0.01, and ^####^ *p* < 0.0001 vs. Control group; ** *p* < 0.01, *** *p* < 0.001, and **** *p* < 0.0001 vs. PI + TSLP group. The original uncropped Western blot images are shown in Figure S2.

Considering that kaempferol derivatives showed the strongest inhibitory activity in earlier experiments, the three most potent candidates (K, K-7-G, and K-7-R) were selected for further mechanistic investigation. KEGG pathway enrichment analysis indicated that JAK/STAT signaling may regulate IL-4 and IL-13 transcription. To further explore this finding, the expression and phosphorylation status of JAK2 and its downstream effectors (STAT3, STAT5, and STAT6) were examined after flavonoid treatment. Specifically, stimulation with PI significantly elevated the phosphorylation levels of STAT3 and STAT6 by 2.1-fold (*p* < 0.0001) and 1.4-fold (*p* < 0.01), respectively, compared with the blank group. In contrast, TSLP alone barely induced the phosphorylation of these two proteins. These results indicate that the activation of STAT3 and STAT6 is primarily mediated by PI, even though the tested compounds were able to reduce the phosphorylation level of STAT3. Further observations revealed that TSLP was capable of inducing STAT5 phosphorylation. Compared with the blank group, TSLP alone increased STAT5 phosphorylation by 1.3-fold. Upon co-stimulation with PI, both JAK2 and STAT5 were significantly activated, with their phosphorylation levels elevated by 1.4-fold (*p* < 0.01) and 1.6-fold (*p* < 0.05), respectively. These results suggest that TSLP preferentially activates the JAK2/STAT5 pathway without evident induction of STAT3 or STAT6, supporting a distinct signaling pattern of TSLP. Notably, intervention with K, K-7-G, or K-7-R markedly reduced phosphorylation of JAK2 and STAT5 compared with the model group (Figure 5A–E), suggesting that these flavonoids exerted anti-inflammatory effects by targeting the TSLP-mediated JAK2/STAT5-specific signaling pathway. To further validate this mechanism, HuT78 cells were pretreated with JAK2 and STAT5 inhibitors before TSLP stimulation. Consistent with the flavonoid results, pharmacological blockade of the JAK2/STAT5 pathway significantly reduced IL-4 and IL-13 secretion (Figure 5F,G).

### 2.6. Effects of K-7-G on Cytokines and Chemokines

**Figure 6:**
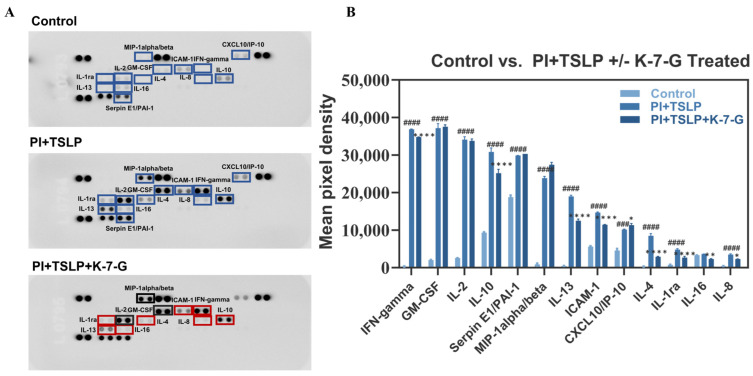
K-7-G treatment reduces pro-inflammatory cytokine secretion in HuT78 cell culture supernatants. Cytokine array analysis of culture supernatants. (**A**) Representative array blots. Blue boxes indicate cytokines significantly upregulated in the PI + TSLP (vs. control); red and black boxes indicate cytokines that are downregulated and show no significant change after K-7-G treatment (vs. PI + TSLP), respectively. (**B**) Mean pixel density of the protein cytokine array (*n* = 2, each group). Data are presented as mean ± SEM. Two-way ANOVA with Tukey’s test was used for multiple comparisons. ^###^ *p* < 0.001 and ^####^ *p* < 0.0001 vs. Control group; * *p* < 0.05, ** *p* < 0.01, and **** *p* < 0.0001 vs. PI + TSLP group.

Based on integrated analysis of multiple preliminary assays, K-7-G was selected for subsequent studies. To evaluate its broader immunomodulatory effects beyond IL-4 and IL-13, cytokine array analysis was performed on culture supernatants from TSLP-stimulated HuT78 cells (Figure 6A,B). TSLP stimulation markedly enhanced the secretion of multiple cytokines; among these, IFN-γ, IL-13, and IL-4 showed the greatest induction (127.5-, 42.9-, and 30.9-fold, respectively), along with MIP-1α/β, GM-CSF, IL-2, IL-8, IL-1ra, IL-10, ICAM-1, CXCL10/IP-10, Serpin E1/PAI-1, and IL-16. Compared with the model group, K-7-G treatment significantly reduced IL-4, IL-1ra, IL-16, IL-8, IL-13, ICAM-1, IL-10, and IFN-γ levels, with IL-4 and IL-13 decreased by 64.9% and 32.4%, respectively. In contrast, K-7-G exerted no significant inhibitory effects on MIP-1α/β, GM-CSF, or IL-2, despite their robust upregulation by TSLP (29.5-, 18.5-, and 13.3-fold, respectively), suggesting a selective rather than broad-spectrum immunomodulatory profile. These findings indicate that K-7-G preferentially targets pathologically elevated type 2 cytokines, most notably IL-4 and IL-13, thereby demonstrating precision immunoregulatory activity with therapeutic relevance to AD.

### 2.7. K-7-G Alleviated DNCB-Induced AD-like Skin Symptoms in Mice

**Figure 7:**
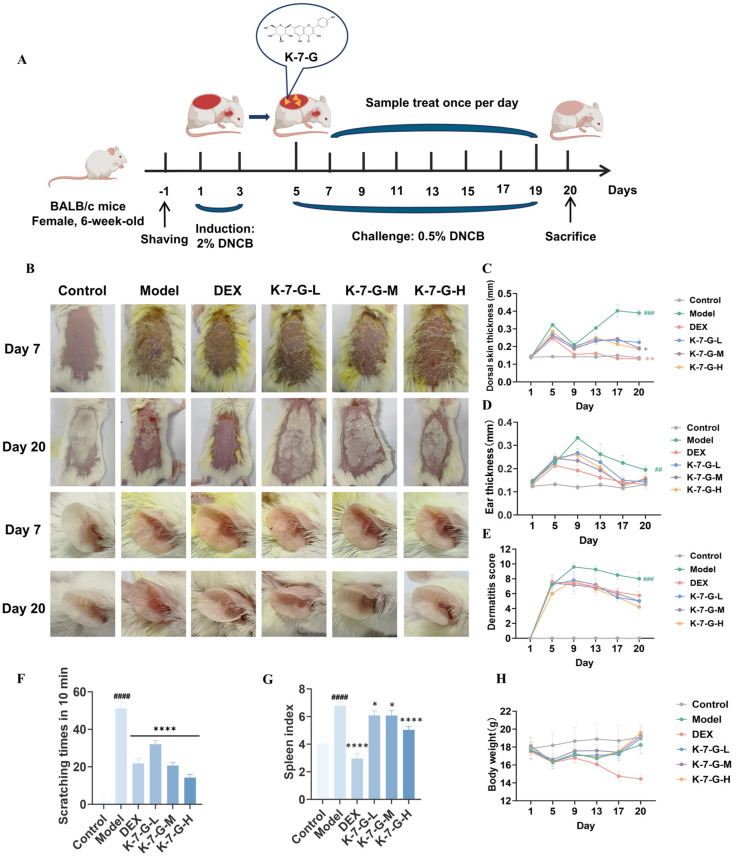
K-7-G alleviates clinical symptoms of DNCB-induced AD mouse model. (**A**) Schematic of the experimental design. (**B**) Typical images of dorsal skin and ears on Day 20. (**C**) Dorsal skin was measured (*n* = 5). (**D**) Ear thickness was determined (*n* = 5). (**E**) Dermatitis scores were evaluated (*n* = 5). (**F**) Scratching frequency was evaluated (*n* = 5). (**G**) The spleen index was evaluated (*n* = 5). (**H**) Body weight changes (*n* = 5). Data are presented as mean ± SEM. One-way ANOVA with Dunnett’s test was used for multiple comparisons. ^##^ *p* < 0.01, ^###^ *p* < 0.001, and ^####^ *p* < 0.0001 vs. Control group; * *p* < 0.05, ** *p* < 0.01, and **** *p* < 0.0001 vs. Model group.

To investigate the in vivo therapeutic potential of K-7-G, this study established a DNCB-induced AD model in BALB/c mice (Figure 7A). The results demonstrated that K-7-G alleviated erythema, edema, and lichenification on the dorsal skin and ear, reduced the dermatitis score, and decreased scratching frequency (Figure 7B–F). Furthermore, the DNCB-treated mice exhibited a marked rise in spleen weights, indicating inflammation and swelling of the spleen. Although DEX treatment reduced both body weight and spleen weight of mice, the reduction exceeded the normal range. In contrast, all K-7-G-treated groups showed significantly reduced spleen weights, which approximated the levels of the control group; additionally, topical application of K-7-G caused no significant change in body weight (Figure 7G,H). These results indicate that K-7-G effectively alleviates DNCB-induced atopic dermatitis-like symptoms in mice and exhibits a favorable safety profile.

### 2.8. K-7-G Alleviates Skin Histopathology in DNCB-Induced AD Mouse

**Figure 8:**
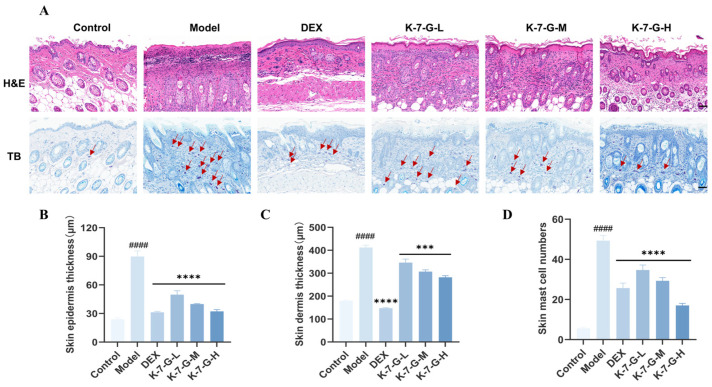
Effect of K-7-G on histological alterations in DNCB-induced AD skin in BALB/c mice. (**A**) H&E staining and TB staining of mouse skin (red arrows indicate mast cells; scale bar: 50 μm). (**B**,**C**) Quantification of epidermal and dermal thickness (*n* = 3). (**D**) Mast cell infiltration was quantified as the average count across five toluidine blue-stained fields (*n* = 3). Data are presented as mean ± SEM. One-way ANOVA with Tukey’s test was used for multiple comparisons. ^####^ *p* < 0.0001 vs. Control group; *** *p* < 0.001 and **** *p* < 0.0001 vs. Model group.

Histopathological evaluation was conducted via H&E and TB staining to further explore the effects of K-7-G on AD-associated pathological features in mice. H&E staining demonstrated that mice induced by DNCB exhibited obvious epidermal and dermal thickening, which was significantly attenuated by K-7-G treatment in the dorsal skin (Figure 8A–C). Similarly, TB staining showed that a large number of mast cells infiltrated the skin tissues in the model group, while the number of infiltrating mast cells in the K-7-G treatment group was significantly reduced (Figure 8D). These results suggest that K-7-G can effectively improve the key histopathological features of the AD model in mice.

### 2.9. K-7-G Reduces Skin Pro-Inflammatory Mediators

**Figure 9:**
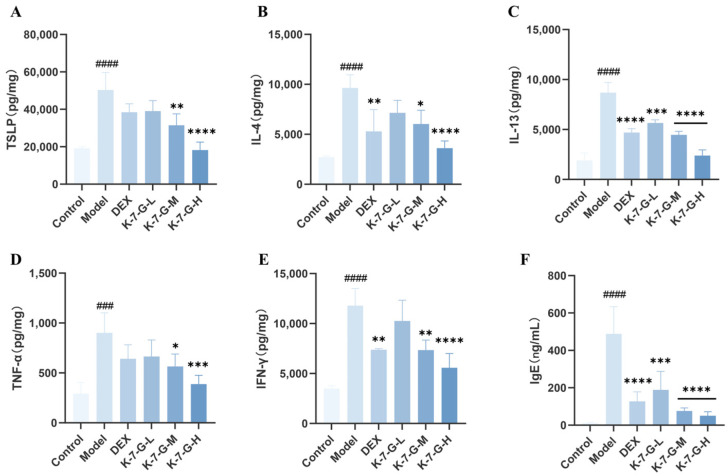
K-7-G suppresses DNCB-induced cytokine expression in BALB/c mice. Mice were euthanized on day 20 for skin biopsy and serum collection. (**A**–**E**) Th1 and Th2 cytokine levels were quantitatively analyzed via ELISA (*n* = 3). (**F**) Quantitative assessment of serum IgE level was conducted through ELISA (*n* = 3). Data are presented as mean ± SEM. One-way ANOVA with Tukey’s test was used for multiple comparisons. ^###^ *p* < 0.001 and ^####^ *p* < 0.0001 vs. Control group; * *p* < 0.05, ** *p* < 0.01, *** *p* < 0.001, and **** *p* < 0.0001 vs. Model group.

On day 20, the cytokine expression and serum IgE levels were measured. The DNCB-exposed group exhibited significant elevation of TSLP and Th2 cytokines (IL-4, IL-13). Conversely, K-7-G administration demonstrated dose-dependent suppression of these pro-inflammatory mediators in AD-like skin conditions (Figure 9A–C). Meanwhile, compared with the control group, DNCB-induced mouse models exhibited a significant upregulation of Th1 cytokines, including TNF-α and IFN-γ (Figure 9D,E), and K-7-G treatment significantly reduced their levels. These results indicate that K-7-G modulates both Th1- and Th2-associated cytokine production in DNCB-induced mice, thereby alleviating AD. Furthermore, K-7-G-treated mice exhibited significantly lower serum IgE levels than the model group, demonstrating that topical K-7-G effectively suppresses IgE expression (Figure 9F). Notably, in both cytokine inhibition and IgE regulation, the efficacy of high-concentration K-7-G was superior to that of the DEX group, exhibiting more significant biological activity.

### 2.10. K-7-G Restores Skin Barrier Proteins

**Figure 10:**
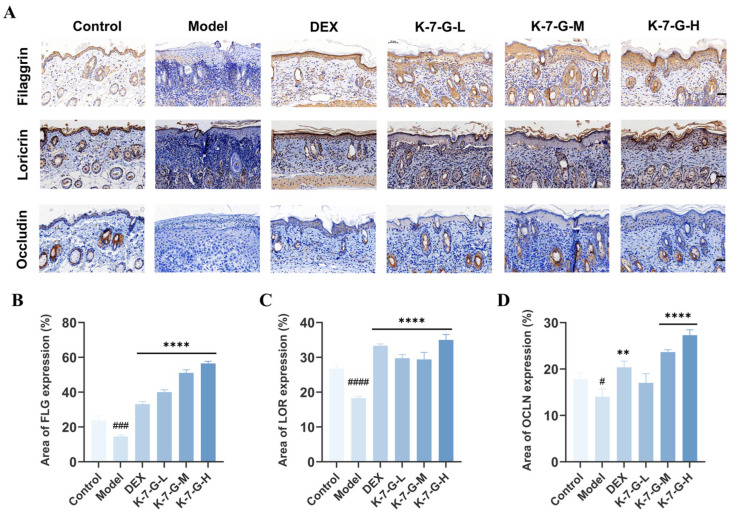
Representative histological sections of mouse skin tissues obtained on Day 20 after immunohistochemical staining are shown. (**A**) Tissues were stained for filaggrin (top panel), loricrin (middle panel), and occludin (bottom panel), respectively. The positive immunohistochemical staining signals of each protein appeared dark brown (scale bar: 50 μm). (**B**–**D**) Quantitative analysis of filaggrin, loricrin, and occludin (*n* = 3). Data are presented as mean ± SEM. One-way ANOVA with Tukey’s test was used for multiple comparisons. ^#^ *p* < 0.05, ^###^ *p* < 0.001 and ^####^ *p* < 0.0001 vs. Control group; ** *p* < 0.01 and **** *p* < 0.0001 vs. Model group.

To elucidate the effect of K-7-G on the levels of epidermal barrier-related proteins in AD-like lesions, immunohistochemistry was performed to evaluate the expression of filaggrin, loricrin, and occludin (Figure 10A,B). K-7-G treatment significantly upregulated the expression of these three key epidermal barrier proteins in DNCB-induced AD-like lesions. These results collectively demonstrate that K-7-G facilitates skin barrier repair by elevating the production of critical epidermal junction proteins, highlighting its therapeutic potential in barrier dysfunction pathologies.

## 3. Discussion

K-7-G is a representative glycoside derivative of kaempferol, which can be isolated from the medicinal plant *Securigera securidaca* [19]. Although K-7-G has been reported to exhibit antioxidant, anti-inflammatory, antiviral, and anti-tumor properties [20], its role in TSLP-mediated allergic inflammation and the structural determinants underlying its activity have remained unexplored. This study systematically examined the inhibitory effects of K-7-G on the TSLP-TSLPR interaction. The mechanism of action was clarified through computational screening, in vitro assays, and in vivo validation in DNCB-induced AD mice.

SAR analysis indicates that the bioactivity of flavonoids, including both cytotoxicity and anti-inflammatory efficacy, depends on the interplay of core skeleton, substitution patterns, and glycosylation. Compound L, lacking a C-3 hydroxyl group, showed stronger cytotoxicity than K and Q, supporting the view that C-3 hydroxyl substitution reduces cytotoxic potential [21]. This suggests a protective role for the C-3 hydroxyl group against nonspecific cellular toxicity, consistent with flavonols generally being less cytotoxic than flavones [22].

Glycosylation represents another critical determinant of flavonoid bioactivity, but its impact is context-dependent. In this study, glycosylation at the C-7 position led to pronounced changes in both cytotoxicity and anti-inflammatory activity. While the aglycones generally exhibited higher cytotoxicity than their corresponding glycosides [23,24], the relationship between glycosylation and anti-inflammatory efficacy proved more complex. Notably, although K displayed moderate inhibition of TSLP-mediated inflammation, its 7-O-glucoside derivative (K-7-G) exhibited markedly superior activity. Further SAR analysis revealed that the identity of the glycosyl moiety at the C-7 position is critical: an O-glucoside (as in K-7-G) demonstrates superior anti-inflammatory efficacy compared to an O-rhamnoside (as in K-7-R) [20], highlighting the importance of the specific sugar moiety in target engagement. This finding appears to contrast with conventional reports that deglycosylation enhances the anti-inflammatory activity of dietary flavonoids, an effect typically attributed to improved bioavailability and stronger suppression of NF-κB/TNF-α pathways [25], as well as studies showing that kaempferol aglycone exerts greater anti-inflammatory effects than its glycosides in certain contexts [20,26]. However, this apparent discrepancy can be resolved by recognizing the target-specific nature of SAR. In the context of TSLP-TSLPR interface disruption, a protein–protein interaction hotspot, the structural requirements for effective inhibition differ fundamentally from those governing conventional anti-inflammatory pathways such as NF-κB. Molecular docking analyses provided mechanistic insights into why K-7-G outperforms its aglycone. The C-7 glucosyl moiety of K-7-G forms multiple specific hydrogen bonds with polar residues on the TSLPR surface, including GLU37, LYS40, and ASP145, interactions that are absent in the aglycone. Therefore, the introduction of the C-7 glucosyl moiety into K-7-G can significantly enhance the binding affinity and thereby improve the inhibitory activity.

The anti-inflammatory potential of flavonoids is also associated with the number and position of hydroxyl groups on their core skeleton [27]. Beyond glycosylation, the anti-inflammatory activity of flavonoids targeting the TSLP pathway is further modulated by these structural features. The hydroxylation pattern on the B ring plays a key role: the presence of a C-3′ hydroxyl group in quercetin and luteolin significantly reduces activity compared with kaempferol derivatives lacking this substitution [28], suggesting steric hindrance or unfavorable electrostatic effects at the binding interface. Additionally, the presence or absence of a C-3 hydroxyl group on the C ring serves as a critical structural determinant, with its absence accounting for the markedly weaker activity of luteolin. Collectively, the synergistic effect of three structural motifs, namely the C-7 O-glucoside substitution on the A ring, the absence of C-3′ hydroxylation on the B ring, and the presence of a C-3 hydroxyl group on the C ring, underlies the potent TSLP inhibitory activity of K-7-G.

TSLP is recognized as a master regulator of Th2-type immune responses, and its interaction with TSLPR initiates downstream JAK/STAT signaling [29,30]. The dendritic cell-mediated Th2 inflammatory cascade triggered by TSLP has been shown to be inhibited through JAK/STAT signaling blockade, with this regulatory mechanism thoroughly validated in existing literature [31]. Transcriptomic analysis confirms that TSLP stimulation can significantly upregulate the JAK/STAT pathway, thereby driving the expression of Th2-type cytokines such as IL-4 and IL-13. This study shows that K-7-G exerts anti-inflammatory effects by inhibiting TSLP-induced JAK2/STAT5 phosphorylation. Notably, K-7-G had no significant effect on the phosphorylation levels of STAT3 or STAT6, suggesting that K-7-G may selectively regulate the TSLP-mediated JAK2/STAT5 signaling pathway. The functional relevance of this pathway was further verified using JAK2 and STAT5 inhibitors, which also reduced the secretion of IL-4 and IL-13, confirming that blocking this signaling cascade is sufficient to inhibit the production of Th2 cytokines.

The pathogenesis of allergic disorders, including AD, is not merely attributed to isolated abnormalities in individual cytokine expression but rather arises from a comprehensive and coordinated dysregulation of the entire inflammatory cytokine network [32]. This network involves not only canonical type 2 mediators (IL-4 and IL-13) but also pro-inflammatory cytokines (IFN-γ) and key chemotactic factors (MIP-1α and MIP-1β), which collectively drive the complex immune imbalance underlying disease progression. In the present study, K-7-G demonstrated a highly selective and targeted immunomodulatory effect rather than a global suppressive action. Specifically, K-7-G significantly attenuated the TSLP-stimulated production of core pathogenic cytokines, including IL-4, IL-13, and IFN-γ, while exerting minimal or no inhibitory effects on other cytokines such as MIP-1α/β, GM-CSF, and IL-2. This pattern of selective regulation suggests that K-7-G may intervene in key signaling nodes within the TSLP-driven cytokine network, rather than causing non-specific broad suppression of immune responses.

AD primarily manifests through visible skin swelling, erythema, and intense itching, all contributing to life quality deterioration [33]. Topical application of K-7-G alleviated clinical symptoms, including reduced ear thickness, decreased dermatitis scores, and lowered frequency of scratching behavior. Histopathological analysis revealed that K-7-G significantly attenuated epidermal and dermal thickening and reduced mast cell infiltration, all of which are key pathological features of AD [34]. AD-associated inflammation is often accompanied by systemic immune dysregulation, and changes in the spleen index can reflect this process; the DNCB-induced increase in spleen weight confirmed the systemic impact of inflammation [35]. Although DEX could alleviate splenic abnormalities, it caused an excessive decrease in body weight and spleen weight along with severe systemic adverse effects [36]. In contrast, K-7-G restored the spleen weight to near-normal levels without affecting body weight, indicating its favorable safety profile. Collectively, these findings demonstrate that flavonoids can effectively alleviate AD-like inflammatory responses with a good safety profile.

The majority of TSLP is secreted by epithelial cells, while dendritic cells and mast cells contribute a smaller amount [37]. As an essential factor in AD pathogenesis, TSLP expression levels have a direct impact on the inflammatory process: increased TSLP expression markedly upregulates pro-inflammatory cytokines and IgE secretion. Furthermore, IL-4 and IL-13 are key downstream effectors of TSLP-mediated Th2-type immune responses and can activate B cells to produce IgE [38,39]. As a core mediator of Th2-type inflammation, IgE amplifies the inflammatory response by activating mast cells, ultimately triggering the characteristic pruritus and skin lesions of AD [40,41]. In contrast, TNF-α and IFN-γ (Th1-type cytokines) function to maintain the chronic state of AD inflammation [42]. Therefore, regulating the expression of cytokines is crucial for mitigating AD pathogenesis. In this study, the levels of TSLP, IL-4, IL-13, TNF-α, IFN-γ, and serum IgE were significantly increased in the AD model group, while K-7-G administration significantly inhibited these mediators. Additionally, K-7-G targeted the core pathology of AD (barrier dysfunction) via the upregulation of filaggrin, loricrin, and occludin. The coupling of anti-inflammatory action with barrier restoration presents a therapeutic advantage over agents that target an isolated pathway. By concurrently suppressing TSLP-driven inflammation and restoring epidermal barrier integrity, K-7-G embodies this mechanistically integrated approach, positioning it as a promising candidate for the holistic management of AD.

This study identifies K-7-G as a natural small-molecule inhibitor of TSLP, furnishing a novel candidate compound for developing highly effective, low-toxicity therapeutics for AD. Nevertheless, this work has certain limitations that also delineate promising avenues for future research. First, the direct binding affinity and kinetic parameters of K-7-G for TSLP or its receptor (TSLPR) require validation via techniques including SPR and ITC to elaborate on their physical interaction modes and precise binding sites. Second, while K-7-G’s therapeutic efficacy has been confirmed in an AD model, its application has not been extended to other TSLP-mediated inflammatory disorders such as allergic asthma [43,44], leaving its broad-spectrum anti-inflammatory activity unconfirmed. Third, given that the present study is largely based on animal models, subsequent research should further validate the efficacy and safety of K-7-G in human skin tissues and clinical samples so as to lay a solid foundation for its clinical translation.

## 4. Materials and Methods

### 4.1. Materials

Luteolin (L), kaempferol (K), and quercetin (Q) were purchased from Shanghai Titan Scientific Co., Ltd. (Shanghai, China). Luteolin-7-O-glucoside (L-7-G), luteolin-7-O-neohesperidoside (L-7-N), and kaempferol-7-O-rhamnoside (K-7-R) were purchased from BioBioPha Co., Ltd. (Kunming, China). Kaempferol-7-O-glucoside (K-7-G) and quercetin-7-O-rhamnoside (Q-7-R) were purchased from Chengdu Desite Biotechnology Co., Ltd. (Chengdu, China). All compounds used had a purity greater than 98%. Human recombinant TSLP, PMA, and ionomycin were purchased from Sigma Aldrich (St. Louis, MO, USA). STAT3, p-STAT3, p-STAT5, STAT6, p-STAT6, JAK2, p-JAK2, and GAPDH antibodies were obtained from Beyotime Biotechnology Co., Ltd. (Shanghai, China). STAT5 antibody was obtained from MedChemExpress (Monmouth Junction, NJ, USA).

### 4.2. Cell Culture and Viability

The HuT78 cell line (BNCC359882) was obtained from the BeNa Culture Collection (BNCC, Xinyang, China). Cells were cultured in IMDM (Gibco, Suzhou, China), enriched with 20% heat-inactivated FBS (Vazyme, Nanjing, China) and 1% penicillin-streptomycin antibiotic-antimycotic mixture. Cultures were kept in a humidified incubator at 37 °C with 5% CO_2_, maintaining a cell density of approximately 1 × 10^6^ cells/mL. To assess cytotoxicity, HuT78 cells were plated and treated with a concentration range of 3.12–50 μM of the flavonoids under screening for 36 h. Finally, the CCK-8 assay (Beyotime, Shanghai, China) was employed to determine cell viability.

### 4.3. Molecular Docking Assay

Molecular docking was conducted using AutoDock4 software. The 3D structure of the protein (PDB ID: 5J11) was accessed through the RCSB PDB database. The flavonoids as ligands were acquired from the TCMSP database. A grid (60 × 60 × 60 points, 0.375 Å spacing) was employed in molecular docking simulations to cover potential binding regions between ligands and receptors. Using Discovery Studio, docking results were assessed to analyze ligand–receptor interactions. Both 2D and 3D visualizations of the protein-ligand complexes were generated.

### 4.4. Transcriptomic Analysis

HuT78 cells (1 × 10^6^ cells/mL) were assigned to two experimental conditions: the control group and the model group, with the model group being exposed to PI [PMA (100 ng/mL) + ionomycin (1 μg/mL)] and TSLP (100 ng/mL) for 24 h. Samples were obtained and preserved at −80 °C for subsequent transcriptomic analysis, ensuring three biological replicates per group. To evaluate heterogeneity within the transcriptomic sequencing datasets between the two groups, PCA plots were utilized. DEGs were identified with the thresholds of |log_2_FC| > 1 and FDR < 0.05. Additionally, the identified transcriptomic DEGs underwent GO functional annotation and KEGG analysis.

### 4.5. RT-qPCR Analysis

Total RNA was extracted from HuT78 cells using an RNA extraction solution (Wuhan Savier, Wuhan, China). RNA concentration and purity were assessed with a NanoDrop ND-1000 spectrophotometer (Thermo Fisher Scientific, Wilmington, DE, USA). cDNA was synthesized by reverse transcription using the SweScript All-in-One RT SuperMix (Wuhan Savier, Wuhan, China). qRT-PCR was performed with 2× Universal Blue SYBR Green qPCR Master Mix (Wuhan Savier, Wuhan, China) on a Bio-Rad CFX Connect system. Melting curve analysis was performed to confirm amplification specificity. GAPDH was used as the internal control, and relative expression levels of IL-4 and IL-13 were calculated using the 2^−ΔΔCt^ method. Primer sequences are listed in Table S2.

### 4.6. ELISA Assay

HuT78 cells (1 × 10^6^ cells/mL) were pretreated with flavonoids (0.78, 3.12, and 12.50 μM) or JAK2/STAT5 inhibitors (50 μM) for 12 h. Following stimulation with PI + TSLP for 24 h, the concentrations of IL-4 and IL-13 were assessed by ELISA kits (Signalway Antibody, College Park, MD, USA). Simultaneously, tissue and blood materials were collected. Blood samples were processed by centrifugation at 3500 rpm for 10 min to prepare serum, while all samples were stored at −80 °C. TSLP, IL-4, IL-13, TNF-α, and IFN-γ levels in skin tissues and total IgE concentration in serum were quantified by ELISA kits (Jonlnbio, Shanghai, China).

### 4.7. TSLP-TSLPR Binding Inhibition Assay

ELISA detection was carried out with Ni-NTA HisSorb 96-well plates (Qiagen, Hilden, Germany). Each well was incubated with 100 μL of hTSLPR-His (0.125 μg/mL) for 2 h. The wells were washed twice, then treated with 200 μL of blocking buffer for 2 h of incubation. After removing the blocking buffer, 50 μL of flavonoid compounds in PBST and 50 μL of hTSLP-Fc (0.5 nM) were introduced into each well and incubated overnight at 4 °C. After three washes with PBST to remove unbound hTSLP-Fc, 100 μL of goat anti-mouse IgG Fc antibody (1:10,000) was added and incubated for 1 h at room temperature. Following five washes, 200 μL of OPD substrate solution was added and incubated for 30 min in the dark. Then, 50 μL of 1.0 mol/L HCl was added to stop the reaction, and absorbance at 450 nm was read using a microplate reader.

### 4.8. Western Blotting Analysis

To assess how flavonoids affect TSLP-driven downstream signaling in HuT78 cells, Western blotting was performed. After preincubating with the compounds for 6 h, cells were stimulated with TSLP for 30 min or 1 h, with or without PI. For protein extraction, IP buffer supplemented with both protease and phosphatase inhibitors was used, with concentrations quantified by BCA assay. Equal amounts of protein (40 μg) were separated via SDS-PAGE (Adamas-Life, Shanghai, China) and transferred to PVDF membranes. The membranes were treated overnight with primary antibodies (1:1000) at 4 °C, then incubated for 1 h with secondary antibodies (1:1000). Detection of immunoreactive bands relied on the BeyoECL Plus kit, with imaging carried out using a fully automated chemiluminescent system. Band intensities were analyzed quantitatively through ImageJ software.

### 4.9. Cytokine Array Assay

The cytokine expression levels in HuT78 cells were assessed using the ARY005B Antibody Array Kit (R&D Systems, Minneapolis, MN, USA). The antibody array PVDF membrane was placed on a shaker and blocked with blocking buffer at room temperature for 1 h, followed by the addition of 700 μL mixture of cell supernatant and 1× detection antibody working solution, and incubated overnight on a shaker at 4 °C. The membrane was then washed three times with 1× wash working solution (10 min per wash). After adding 1× HRP working solution, the membrane was incubated for 30 min on a shaker at room temperature and washed using the same method, followed by incubation with chemiluminescent substrate mixture for 1 min at room temperature. The signal intensity was measured with the ChemiScope 6300 system, and grayscale analysis was performed using HLImage++ software (Version 1.9.1) to quantify the expression levels of each factor.

### 4.10. Animals

BALB/c mice (female, 18–20 g, 6 weeks old) were purchased from Hubay Collaborative Biomedical Technology Co., Ltd. (Changsha, China). and housed under SPF conditions. Temperature and humidity were kept between 20 and 25 °C and 40–60%, respectively, while a strict 12 h light-dark cycle was followed to limit environmental interference. All experiments were approved by the Ethics Committee of Jiangsu Institute of Parasitic Diseases (Wuxi, China), approval number JIPD-IACUC-2025049.

### 4.11. DNCB-Induced AD in Mice

Six groups were randomly formed with thirty healthy mice, with five mice in each group. The groups are as follows: untreated control (Control), DNCB (Model), DEX (Positive control), low-dose K-7-G (0.1%, K-7-G-L), medium-dose K-7-G (0.5%, K-7-G-M), and high-dose K-7-G (1%, K-7-G-H). Prior to sensitization, dorsal hair was shaved with a razor. On Days 1 and 3, 2% DNCB was administered as 150 μL to the back skin and 20 μL to the ear. Five days post-depilation, 0.5% DNCB was topically applied to both areas four times weekly for 15 days. From Day 7, the respective test agents were applied topically to the back (80 μL) and the ear (10 μL) daily for 13 straight days. On the last experimental day, mice were anesthetized using isoflurane, with blood, dorsal skin, and spleen samples collected for subsequent analyses. During the experiment, the mice were weighed every three days and their body weights were recorded accordingly. Following a previously reported protocol, dorsal skin lesion severity in AD mice was evaluated using four criteria (erythema, edema/papules, desquamation/scaling, and lichenification). Dorsal skin and right ear thickness were quantified with an electronic caliper. To evaluate AD-like behavioral alterations, on Day 20, immediately after DNCB sensitization, scratching behavior directed at the nose, ears, and dorsal skin was recorded over a 10 min period. On the same day, spleens were collected and weighed to calculate the spleen index, defined as:

Spleen Index (mg/g) = Wet Spleen Weight (mg)/Mouse Body Weight (g)

### 4.12. Histopathological Analysis of Skin Lesions

For assessing epidermal thickening, each mouse’s dorsal skin samples were gathered on Day 20 (*n* = 3 per group). These samples were preserved in 4% PFA and then subjected to paraffin embedding for further processing. Subsequently, the samples were sectioned into 6-μm-thick slices and subjected to H&E staining, Toluidine Blue staining, and immunohistochemical staining for filaggrin, loricrin, and occludin. All stained sections were observed under a 20× microscope.

### 4.13. Statistical Analysis

GraphPad Prism software (version 10.1.2) was used for all statistical analyses and graph generation. Data are presented as mean ± SEM from at least three independent replicates unless otherwise specified. Data distribution was assessed using the Shapiro–Wilk normality test, and homogeneity of variances was analyzed using Levene’s test prior to statistical comparisons. For comparisons involving multiple groups, one-way ANOVA or two-way ANOVA was used, followed by appropriate post hoc tests: Dunnett’s test for comparisons against a control or model group, and Tukey’s test for all pairwise comparisons. For transcriptomic analysis, DEGs were identified using the Wald test implemented in DESeq2, with FDR correction by the Benjamini–Hochberg method. Genes meeting the thresholds of |log_2_FC| > 1 and FDR < 0.05 were considered significantly differentially expressed. Statistical significance was considered as follows: ^#^ *p* < 0.05, ^##^ *p* < 0.01, ^###^ *p* < 0.001, and ^####^ *p* < 0.0001 vs. Control group; * *p* < 0.05, ** *p* < 0.01, *** *p* < 0.001, and **** *p* < 0.0001 vs. Model group.

## 5. Conclusions

In conclusion, this study identifies K-7-G as a naturally derived TSLP inhibitor. Its bioactivity depends on three core structural features: a C-7 glucosyl group on ring A, a C-3 hydroxyl group on ring C, and the absence of a 3′ hydroxyl group on ring B. Mechanistically, K-7-G inhibits the binding of TSLP to TSLPR, selectively suppresses TSLP-induced JAK2/STAT5 signaling, and significantly downregulates the expression of the Th2 cytokines. In addition, this study confirmed that topical K-7-G administration effectively downregulates pro-inflammatory cytokine expression and restores skin barrier protein levels, thereby exerting dual anti-inflammatory and skin barrier repair effects. Moreover, it exhibits superior safety compared with glucocorticoids. Thus, K-7-G represents a promising natural candidate for treating atopic dermatitis and potentially other TSLP-mediated inflammatory diseases.

## Data Availability

The data is contained within the article.

## Supplementary Materials

The following supporting information can be downloaded at https://www.mdpi.com/article/10.3390/ph19040580/s1, Table S1: Substituents and binding energies of eight flavonoid compounds; Table S2: Primer sequences for qRT-PCR analysis; Figure S1: Schematic diagrams of molecular docking interactions between seven flavonoids and target protein residues; Figure S2: Original uncropped Western blot images.

## Abbreviations

AD	Atopic dermatitis
TSLP	Thymic stromal lymphopoietin
TSLPR	Thymic stromal lymphopoietin receptor
SAR	Structure–activity relationship
K-7-G	Kaempferol-7-O-glucoside
JAK	Janus kinase
STAT	Signal transducer and activator of transcription
IL-4	Interleukin-4
IL-13	Interleukin-13
TNF-α	Tumor Necrosis Factor-α
IFN-γ	Interferon-γ
IgE	Immunoglobulin E
IL-7Rα	Interleukin-7 receptor alpha subunit
DEGs	Differentially expressed genes
PCA	Principal component analysis
GO	Gene ontology
KEGG	Kyoto encyclopedia of genes and genomes
BP	Biological process
MF	Molecular function
CC	Cellular component
DNCB	Dinitrochlorobenzene
DEX	Dexamethasone
H&E	Hematoxylin and eosin
TB	Toluidine blue
PMA	Phorbol 12-myristate 13-acetate
