# Supplementary material for: Kaempferol-7-O-Glucoside Ameliorates Atopic Dermatitis via the TSLP-Mediated JAK2/STAT5 Signaling Axis
Source: Pharmaceuticals (Basel). 2026 Apr 4;19(4):580. doi: 10.3390/ph19040580 (PMC13118320; doi:10.3390/ph19040580)
Supplement: Supplementary file 1 [file pharmaceuticals-19-00580-s001.zip › pharmaceuticals-4228785-supplementary.pdf]

Kaempferol-7-O-glucoside Ameliorates Atopic Dermatitis via the TSLP-mediated JAK2/STAT5 Signaling Axis

Xingmei Lan [1], Jing Liu [1], Yijie Shi [1], Yonghua Zhou [2], Cheng Yang [1,*] and Bingtian Zhao [1,*]

[1] Key Laboratory of Synthetic and Biological Colloids, Ministry of Education, School of Chemical and Material Engineering, Jiangnan University, Wuxi 214122, China; lanxingmei999@163.com (X.-M.L.); lj15735649029@163.com (J.L.); 1053230304@stu.jiangnan.edu.cn (Y.-J.S.)

[2] Key Laboratory of National Health Commission on Parasitic Disease Control and Prevention, Jiangsu Provincial Key Laboratory on Parasite and Vector Control, Jiangsu Institute of Parasitic Diseases and Public Health Research Center of Jiangnan University, Wuxi 214064, China.; toxo2001@163.com (Y.-H.Z.)

* Correspondence: btzhao@jiangnan.edu.cn (B.-T.Z.); cyang@jiangnan.edu.cn (C.Y.);
Tel.: +86-186-4333-5239 (B.-T.Z.)

Table S1. Substituents and binding energies of eight flavonoid compounds

**Compounds**	**3'**	**3**	**7**	**Binding energ （kcal/mol）**
Luteolin	OH	H	OH	-6.0
Kaempferol	H	OH	OH	-6.4
Quercetin	OH	OH	OH	-6.5
Luteolin-7-O-glucoside	OH	H	O-glucoside	-7.4
Luteolin-7-O-neohesperidoside	OH	H	O-neohesperidoside	-7.3
Kaempferol-7-O-glucoside	H	OH	O-glucoside	-7.1
Kaempferol-7-O-rhamnoside	H	OH	O-rhamnoside	-7.2
Quercetin-7-O-rhamnoside	OH	OH	O-rhamnoside	-7.3

Table S2. Primer Sequences for qRT-PCR analysis

**Gene**	**Primer**	**Sequence (5'→3')**	**Length (bp)**
GAPDH	Forward	GGAAGCTTGTCATCAATGGAAATC	168
	Reverse	TGATGACCCTTTTGGCTCCC	
IL-4	Forward	GGTCTCACCTCCCAACTGCTTC	135
	Reverse	AGTCTTCTGCTCTGTGAGGCTGTT	
IL-13	Forward	CATGTACTGTGCAGCCCTGGA	86
	Reverse	AGAATCCGCTCAGCATCCTCT	

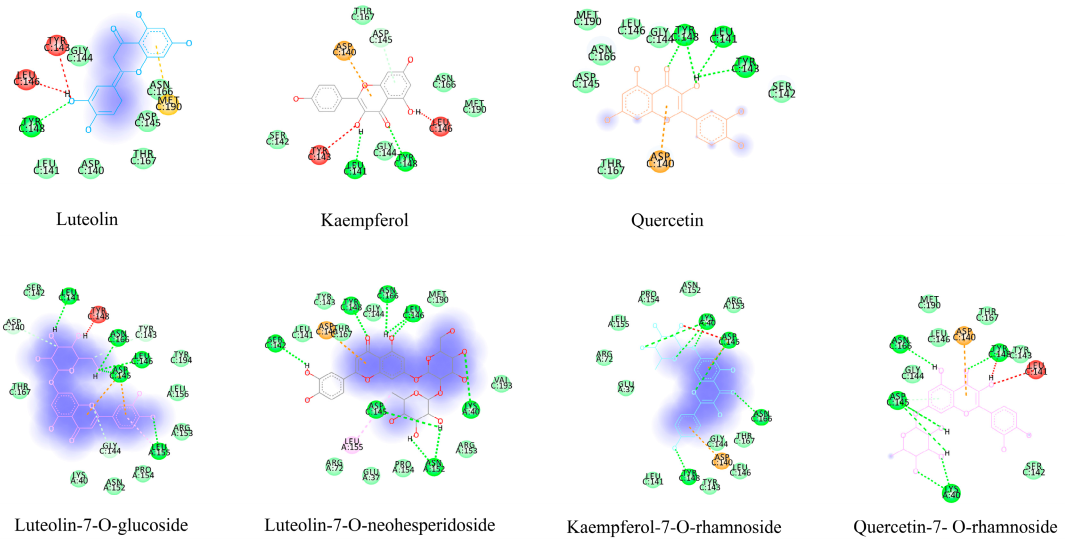

Figure S1. Schematic diagrams of molecular docking interactions between seven flavonoids and target protein residues.

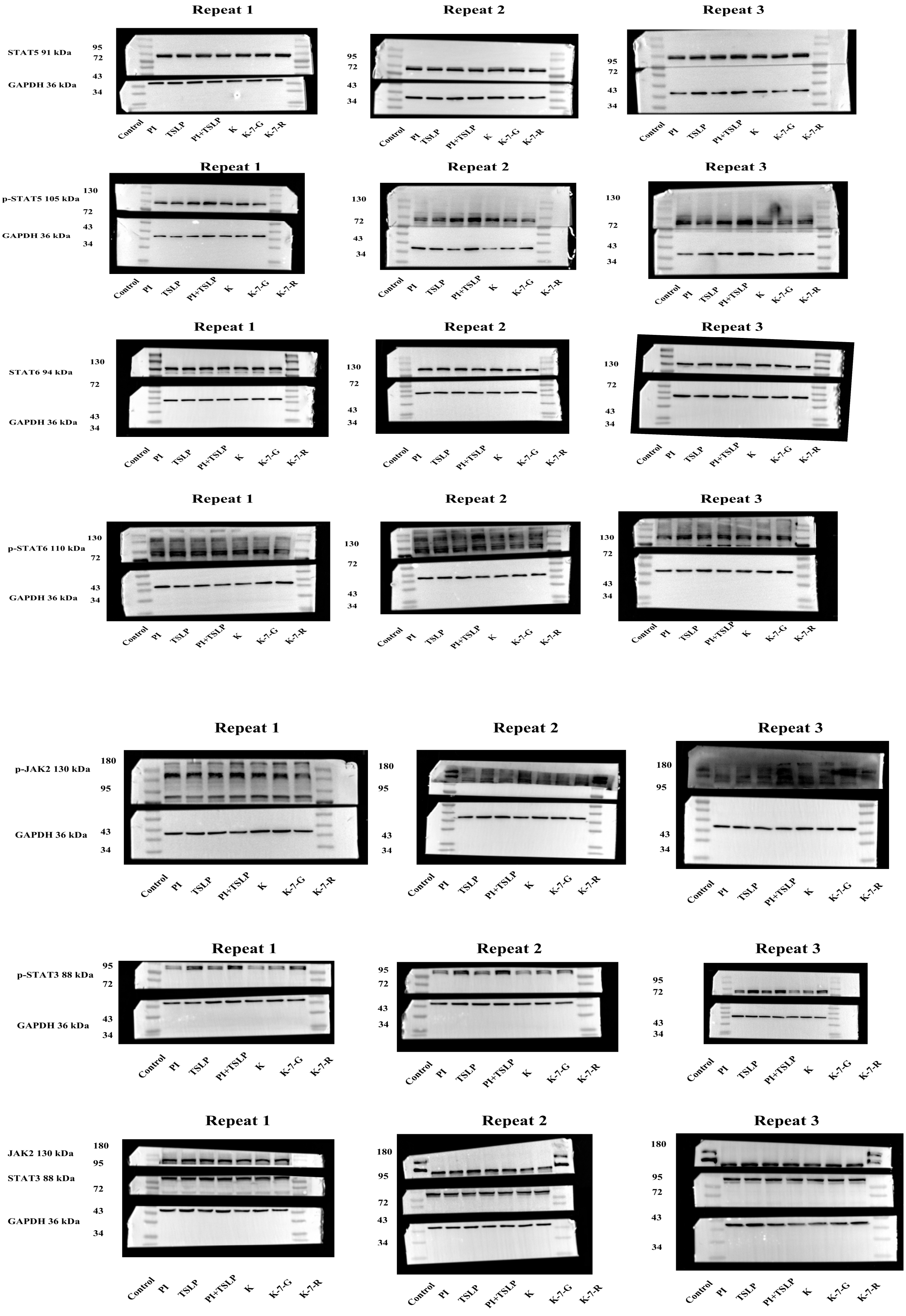

Figure S2. Original uncropped Western blot images.
